# Genomic epidemiology of global *Klebsiella pneumoniae* carbapenemase (KPC)-producing *Escherichia coli*

**DOI:** 10.1038/s41598-017-06256-2

**Published:** 2017-07-19

**Authors:** N. Stoesser, A. E. Sheppard, G. Peirano, L. W. Anson, L. Pankhurst, R. Sebra, H. T. T. Phan, A. Kasarskis, A. J. Mathers, T. E. A. Peto, P. Bradford, M. R. Motyl, A. S. Walker, D. W. Crook, J. D. Pitout

**Affiliations:** 1 Modernising Medical Microbiology Consortium, Nuffield Department of Medicine, John Radcliffe Hospital, University of Oxford, Oxford, United Kingdom; 2 Division of Microbiology, Calgary Laboratory Services, Calgary, Alberta Canada; 3 Department of Pathology and Laboratory Medicine, University of Calgary, Alberta, Canada; 4 Icahn Institute and Department of Genetics and Genomic Sciences, Icahn School of Medicine, Mount Sinai, New York USA; 5 National Institute for Health Research (NIHR) Health Protection Research Unit (NIHR HPRU) in Healthcare Associated Infections and Antimicrobial Resistance, University of Oxford, Oxford, United Kingdom; 6 Division of Infectious Diseases and International Health, Department of Medicine, University of Virginia Health System, Charlottesville, Virginia USA; 7 Office of Hospital Epidemiology, University of Virginia Health System, Charlottesville, Virginia USA; 8 AstraZeneca Pharmaceuticals LP, Waltham Massachusetts, USA; 9 Clinical Microbiology, Merck and Co Inc., Rahway, New Jersey, USA; 10 Department of Microbiology, Immunology and Infectious diseases, University of Calgary, Alberta, Canada; 11 Snyder Institute for Chronic diseases, University of Calgary, Alberta, Canada; 12 Department of Medical Microbiology, University of Pretoria, Pretoria, South Africa

## Abstract

The dissemination of carbapenem resistance in *Escherichia coli* has major implications for the management of common infections. *bla*_KPC_, encoding a transmissible carbapenemase (KPC), has historically largely been associated with *Klebsiella pneumoniae*, a predominant plasmid (pKpQIL), and a specific transposable element (Tn*4401*, ~10 kb). Here we characterize the genetic features of *bla*_KPC_ emergence in global *E. coli*, 2008–2013, using both long- and short-read whole-genome sequencing. Amongst 43/45 successfully sequenced *bla*_KPC_-*E. coli* strains, we identified substantial strain diversity (n = 21 sequence types, 18% of annotated genes in the core genome); substantial plasmid diversity (≥9 replicon types); and substantial *bla*_KPC_-associated, mobile genetic element (MGE) diversity (50% not within complete Tn*4401* elements). We also found evidence of inter-species, regional and international plasmid spread. In several cases *bla*_KPC_ was found on high copy number, small Col-like plasmids, previously associated with horizontal transmission of resistance genes in the absence of antimicrobial selection pressures. *E. coli* is a common human pathogen, but also a commensal in multiple environmental and animal reservoirs, and easily transmissible. The association of *bla*_KPC_ with a range of MGEs previously linked to the successful spread of widely endemic resistance mechanisms (e.g. *bla*_TEM_, *bla*_CTX-M_) suggests that it may become similarly prevalent.

## Introduction

Carbapenemases have emerged over the last 15 years as a major antimicrobial resistance threat in Enterobacteriaceae, many species of which are major human pathogens^1^. They are enzymes with broad-spectrum hydrolytic activity targeting beta-lactams, and commonly associated with additional resistance mechanisms producing cross-resistance to other antimicrobial classes^2^. The *Klebsiella pneumoniae* carbapenemase (KPC) enzyme, encoded by alleles of the *bla*_KPC_ gene, represents one of the five major carbapenemase families, others being the VIM, IMP and NDM metallo-beta-lactamases, and the OXA-48-like oxacillinases^3^.

KPC, unlike the other major carbapenemases, is an Ambler class A enzyme, with a serine in the active site, which hydrolyses penicillins, cephalosporins, aztreonam and carbapenems^4^. At least 18 variants are known, with nucleotide mutations across 20 positions (13 amino acid substitutions), and one variant with a 6 bp deletion (KPC-14, nucleotide positions: 722–727]. *bla*_KPC_ is typically located within a 10 kb mobile transposon (Tn*4401*), most often on conjugative plasmids. In publicly available sequence data, *bla*_KPC_ is mostly found as a single copy on any individual plasmid although it can exist in duplicate (6/133 [5%] KPC plasmid structures with Tn*4401*/*bla*_KPC-2/3_ duplications available in GenBank, March 2017 [Phan HTT, unpublished data]). It has also been described on multiple plasmids within the same isolate, and/or in multiple copies shared amongst the chromosome and plasmids^5, 6^.

The first KPC-producer, a *K. pneumoniae* strain harbouring *bla*_KPC-2_, was identified in 1996 in the eastern USA; since then, KPC-2 and KPC-3 (H272Y [C814T] with respect to KPC-2) have become widespread, and entrenched in endemic hotspots in the USA, Greece, Israel, China and Latin America^7, 8^. KPC-3 confers a 4-fold increase in ceftazidime resistance compared with KPC-2^9^. The other variants remain relatively rare in published surveys. The spread of the epidemic *K. pneumoniae* lineage, ST258, is thought to have contributed significantly to global *bla*_KPC-2_/*bla*_KPC-3_ dissemination^10^, although these genes have now been observed in several species of Enterobacteriaceae^5, 6^.

Acquired carbapenem resistance in *Escherichia coli* was considered rare as recently as 2010, although the first cases of KPC-*E. coli* were observed as early as 2004–2005 in Cleveland (n = 1, KPC-2^11^), New York City (n = 2, KPC-2), New Jersey, USA (n = 1, KPC-3)^12^, and Tel Aviv, Israel (n = 4, KPC-2)^13, 14^. No apparent epidemiological links between any of these cases were identified. Genotyping was limited at this time, but supported diversity being present in both host *E. coli* and *bla*_KPC_ plasmid backgrounds. Since then, direct, plasmid-mediated transfer of *bla*_KPC_ into *E. coli* within human hosts has been described^15^, and clusters of KPC-*E. coli* have been identified in several locations, from China to Puerto Rico^16, 17^, and in the context of clinical infections^16, 17^, asymptomatic colonization^18^ and the environment^19^.

More recently there has been concern around *bla*_KPC_ in *E. coli* sequence type (ST) 131, a globally disseminated, clinically successful strain^20–22^. Notably, the H30R/C1 (fluoroquinolone-resistant) and H30Rx/C2 (fluoroquinolone and extended-spectrum cephalosporin-resistant) ST131 sub-lineages have previously emerged in association with particular drug resistance mechanisms, including the extended-spectrum beta-lactamase (ESBL) gene, *bla*_CTX-M-15_ (clade C2)^23, 24^. Given the high rates of ST131 community and healthcare-associated infections^25^, and its capacity for asymptomatic gastrointestinal colonisation^26^, a stable association of ST131 with *bla*_KPC_ could have important consequences for the management of *E. coli* infections^14^.

Despite these concerns, there are limited detailed molecular epidemiological data investigating the genetic structures associated with *bla*_KPC_ in *E. coli* and the extent to which these may have been shared amongst Enterobacteriaceae. Here we used short-read (Illumina) and long-read (PacBio) sequencing to investigate 43 *bla*_KPC_-positive *E. coli* isolates obtained consecutively from global surveillance schemes (67 participating countries, 2008–2013), fully resolving the *bla*_KPC_-containing plasmids in 22 cases, and comparing these data with other *bla*_KPC_ plasmid sequences.

## Results

### Global *bla*_KPC_-*E. coli* strains are diverse, even within the most prevalent ST, ST131, with evidence for local transmission

45 isolates were obtained from 21 cities in 11 countries across four continents (2010–2013; previous laboratory typing results summarized in Table S1). One isolate was *bla*_KPC_-negative on whole-genome sequencing (WGS; ecol_252), potentially having lost *bla*_KPC_ during storage or sub-culture in the intervening time period between when the original typing was undertaken and the subsequent DNA extraction and preparation for WGS. For one isolate the WGS data were inconsistent with the lab typing results (ecol_451), likely representing a laboratory mix-up; ecol_252 and ecol_451 were therefore excluded from subsequent analyses. The other 43 isolates were successfully sequenced (for quality metrics see Table S1). Amongst these 43 isolates, twenty-one different *E. coli* STs were represented (Table 1; predicted *in silico* from WGS), including: ST131 [n = 16], ST410 [n = 4], ST38 [n = 3], ST10, ST69 [n = 2 each] (remaining isolates singleton STs).

**Table 1:** Plasmid replicon families present by ST, using the PlasmidFinder database^58^.

Inc type	Sequence type (number of isolates)																					Total number of isolates [number of *bla*_KPC_ plasmids]	*p*
	10 (n=2)	38 (3)	44 (1)	69 (2)	101 (1)	131 (16)	155 (1)	167 (1)	182 (1)	224 (1)	297 (1)	354 (1)	361 (1)	393 (1)	410 (4)	428 (1)	540 (1)	648 (1)	744 (1)	1193 (1)	1431 (1)		
A/C2		1	1[1]							1				1[1]								4[2]	0.05
B/O/K/Z						1																1[0]	1
FIA	1	1	1		1	15		1				1		1	2			1		1		26[0]	**0.001**
FIB	2	1		2	1	11	1	1				1		1	4		1	1	1	1		29[0]	0.14
FII	1	2	1	1	1	12[2] ^a,b^		1				1		1	4[2]	1	1[1] ^c^	1	1	1		30[5]^a,b,c^	0.34
FIC(FII)						1	1															2[0]	0.67
HI1b	1																					1[0]	0.47
HI2+HIA2						1																1[0]	1
I1	1	2			1		1								2		1					8[0]	**0.02**
I2							1															1[0]	0.67
L/M		1[1]									1											2[1]	0.26
N	1[1]			1	1[1]	3[3]		1	1				1		4[2]			1[1]	1		1	16[8]	**0.009**
P						1[1]	1								2							4[1]	0.47
Q1				1		2[1]								1					1	1		6[1]	0.31
R						2[1]																2[1]	1
U				1[1]								1[1]										2[2]	0.21
X1																			1			1	0.37
X3						3																3[0]	1
X4						4																3[0]	0.98
Y										1												1[0]	0.37
col	1	1	1	1		12[5] ^d^	1[1]				1[1]				4					1	1	24[7]^d^	**0.04**
p0111												1											0.37

Undmbers in square brackets represent the known subset of *bla*_KPC_ plasmids in each cell. Exact test compares presence/absence of each Inc type by ST. The replicon type specifically associated with *bla*_KPC_ could not be evaluated in 15 isolates, due to limitations of the assemblies.

^a^one multi-replicon plasmid also containing IncFIA.

^b^one multi-replicon plasmid also containing IncFIA and IncR.

^c^one multi-replicon plasmid also containing IncFIB.

^d^one multi-replicon plasmid also containing *repA*.

**Figure 1:**
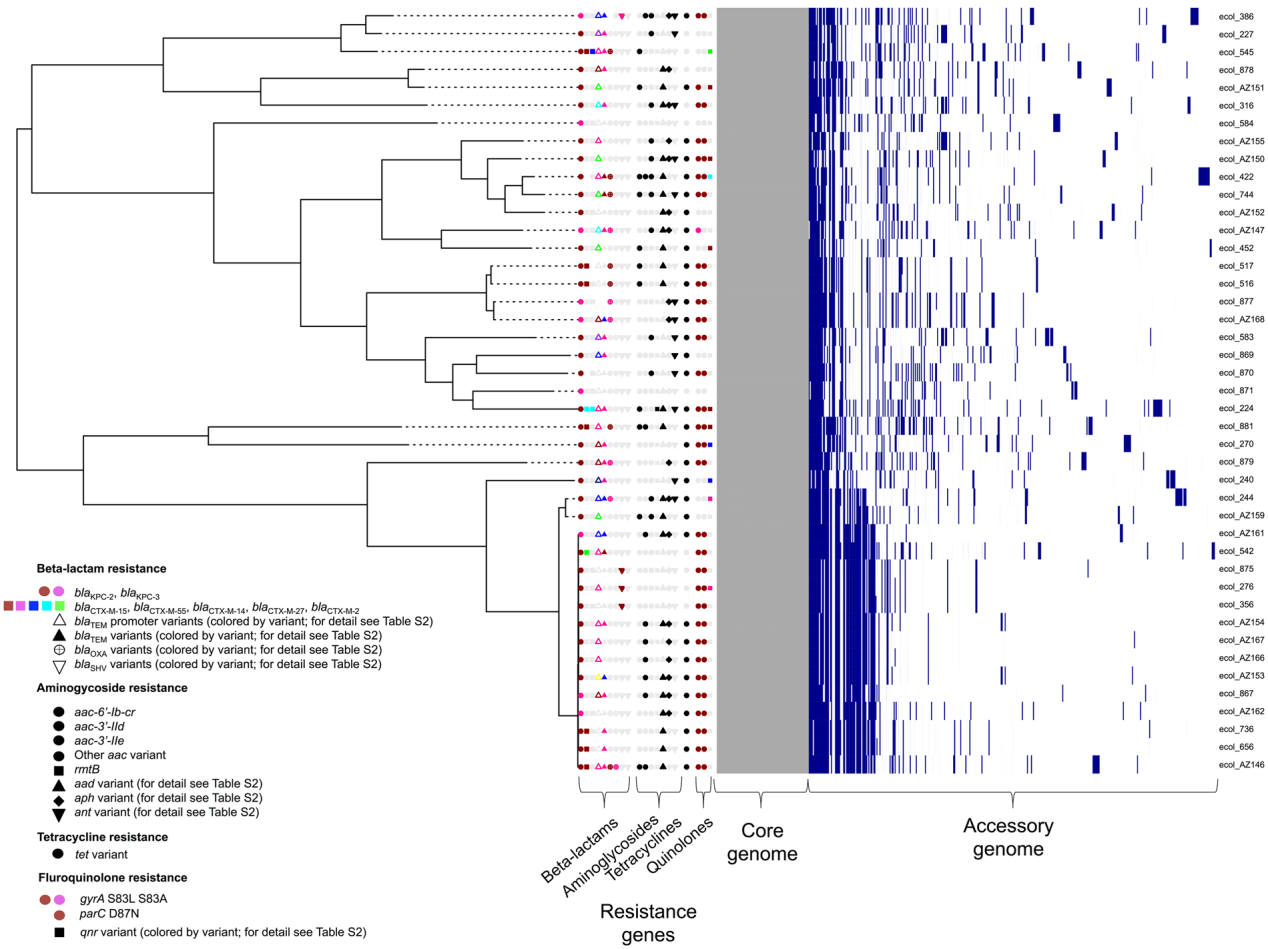
Phylogeny of KPC-*Escherichia coli* identified from global carbapenem resistance surveillance schemes, 2008–2013. Panels to the right of the phylogeny represent common resistance gene mechanisms (full details of resistance gene typing in Table S2), core and accessory genome components. For the accessory genome panel, blue represents annotated regions that are present, and white those that are absent.

Of 16,053 annotated open reading frames (ORFs) identified across all KPC-*E. coli* isolates, only 2,950 (18.4%) were shared in all isolates (“core”), and a further 222 (1.4%) in 95- < 100% of isolates (“soft core”^27^). At the nucleotide level there were 213,352 single nucleotide variants (SNVs) in the core genome, consistent with the previously observed species diversity^28^. Resistance gene profiles also varied markedly between strains, with some harbouring several beta-lactam, aminoglycoside, tetracycline and fluoroquinolone resistance mechanisms (e.g. ecol_224) and others containing *bla*_KPC_ only (e.g. ecol_584; Fig. 1). For the 16 KPC-ST131 strains, 4,071/7,910 (51%) ORFs were core, with 6,778 SNVs across the core genome of these isolates, again consistent with previous global studies of ST131 diversity^23, 24^ (Figure S1). Accessory genomes were highly concordant for some (e.g. ecol_356/ecol_276/ecol_875), but not all (e.g. ecol_AZ159/ecol_244) isolates that were closely related in their core genomes, supporting highly variable evolutionary dynamics between core and accessory genomes (Fig. 1). The geographic distribution of isolates closely related in both the core and accessory genomes supports local (e.g. ecol_AZ166, ecol_AZ167 [ST131, Beijing, China]) transmission of particular KPC-*E. coli* strains. The homology of genetic flanking motifs around the *bla*_KPC_ genes in these closely related isolate pairs would also be consistent with this hypothesis, and less consistent with multiple acquisition events of *bla*_KPC_ within the same genetic background, especially given the diversity in *bla*_KPC_ flanking sequences observed across the rest of the dataset (see below).

### *bla*_KPC_ genes appear restricted to plasmid contexts in *E. coli* at present, but may exist in multiple copies on single plasmid structures or in high copy number plasmids

Thirty-four isolates (80%) contained *bla*_KPC-2_, and nine isolates (20%) *bla*_KPC-3_. Chromosomal integration of *bla*_KPC_ has been described in other Enterobacteriaceae, *Pseudomonas* and *Acinetobacter* spp. but remains rare^5, 29, 30^. There was no evidence of chromosomal integration of *bla*_KPC_ in either the 18 chromosomal structures reconstructed from long-read sequencing, or based on review of the annotations in the *bla*_KPC_-containing contigs (derived from Illumina *de novo* assemblies) for the other 25 isolates. *bla*_KPC_ alleles were not segregated by ST.

Estimates of *bla*_KPC_ copy number per bacterial chromosome varied between <1 (ecol_879, ecol_881) and 55 (ecol_AZ152). In nine cases this estimate was ≥10 copies of *bla*_KPC_ per bacterial chromosome (ecol_276, ecol_356, ecol_867, ecol_869, ecol_870, ecol_875, ecol_AZ150, ecol_AZ152, ecol_AZ159, Table S2). Six of these isolates contained *bla*_KPC_ in a col-like plasmid context, in two cases the plasmid rep type was unknown, and in one case it was an IncN replicon. Plasmid copy number is associated with higher levels of antibiotic resistance if the relevant gene is located on a high-copy unit. Interestingly, high copy number plasmids are postulated to have higher chances of fixing in descendant cells, as they distribute more adequately by chance and without the requirement for partitioning systems^31^, and of being transferred in any conjugation event, either directly or indirectly^32–34^.

### *bla*_KPC_ and non-*bla*_KPC_ plasmid populations across global KPC-*E. coli* strains are extremely diverse

Plasmid Inc typing revealed the presence of a median of four plasmid replicon types per isolate (range: 1–6; IQR: 3–5), representing wide diversity (Table 1). However, IncN, col, IncFIA and IncI1 replicons were disproportionately over-represented in certain STs (p < 0.05; Table 1). Amongst the 18 isolates that underwent PacBio sequencing, we identified 53 closed, non-*bla*_KPC_ plasmids, ranging from 1,459 bp to 289,903 bp (Table S1; at least four additional, partially complete plasmid structures were present). Of these non-*bla*_KPC_ plasmids, 10 (size: 2,571–150,994 bp) had <70% similarity (defined by percent sequence identity multiplied by proportion of query length demonstrating homology) to other sequences available in GenBank, highlighting that a proportion of the “plasmidome” in KPC-*E. coli* remains incompletely characterized. For the other 43 plasmids, the top match in GenBank was a plasmid from *E. coli* in 35 cases, *K. pneumoniae* in 5 cases, and *Citrobacter freundii*, *Shigella sonnei*, *Salmonella enterica* in 1 case each (Table S3).

Twenty-two *bla*_KPC_ plasmid structures were fully resolved (17 from Pacbio data only, four from Illumina data only, 1 from both PacBio and Illumina data), ranging from 14,029 bp to 287,067 bp (median = 55,590 bp; IQR: 23,499–82,765 bp). These *bla*_KPC_-containing plasmids, and six additional cases where *bla*_KPC_ was identified on a replicon-containing contig, were highly diverse based on Inc typing (Table S1). IncN was the most common type (n = 8/28 type-able *bla*_KPC_ structures; 29%), followed by small, col-like plasmids (n = 6/28 [col-like plasmids with single replicons only]; 21%). Other less common types were: A/C2, FII(k), U (all n = 2); and L/M, P, Q1 and R (all n = 1). Four (14%) *bla*_KPC_ plasmids were multi-replicon constructs, namely: col/*repA*, FIB/FII, FIA/FII, and FIA/FII/R.

### Common IncN plasmid backbones have dispersed globally within *E. coli*

From GenBank, we selected all unique, fully sequenced IncN-*bla*_KPC_ plasmid sequences (Table S4) for comparison, dating from as early as 2005, around the time of the earliest reports of KPC-producing *E. coli*. The plasmid backbones and flanking sequences surrounding *bla*_KPC_ in these 16 plasmid references and a subset of 12 study sequences (see “Methods”) were consistent with multiple acquisitions of two known IncN-Tn*4401*-*bla*_KPC_ complexes in divergent *E. coli* STs: firstly, within a Plasmid-9 (FJ223607, 2005, USA)-like background, and secondly, within a Tn*2*/*3*-like element in a Plasmid-12 (FJ223605, 2005, USA)-like background.

**Figure 2:**
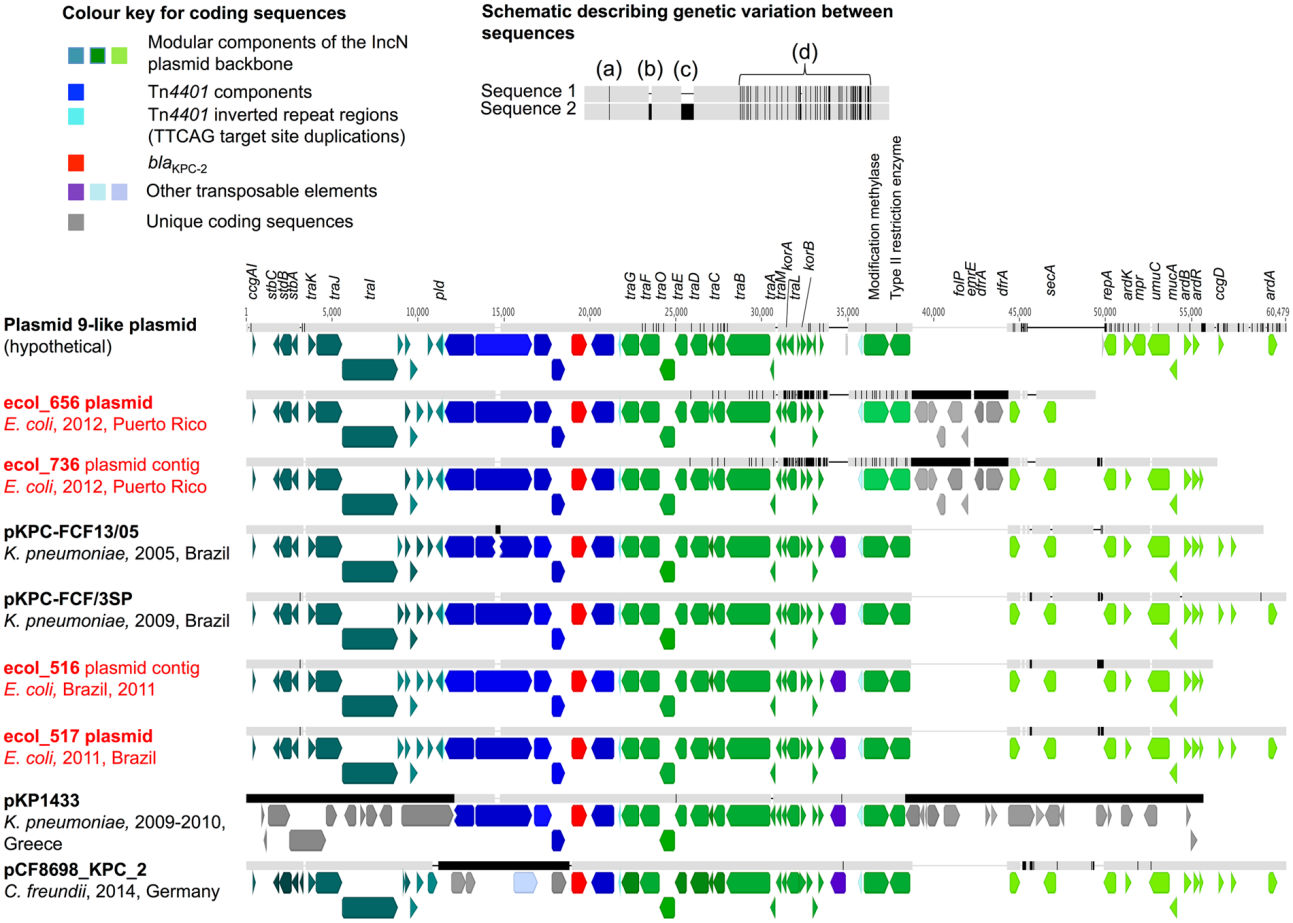
Comparison schematic of FJ223607-like (Plasmid 9-like) IncN plasmids (publicly available; this study), and their geographic origin/dates of isolation. Plasmid sequence names in red are those from this study, derived from PacBio data and closed (ecol_517, ecol_656) or incomplete plasmid structures (ecol_516, ecol_736) derived from Illumina data. Aligned bars adjacent to plasmid names represent plasmid sequences: light grey denotes regions with 100% sequence identity; black represents nucleotide diversity between sequences; and thin lines represent indels. Coding sequences are represented by fat arrows below individual sequence bars and are colour-coded as per the colour key. The inset schematic describing genetic variation between sequences depicts examples of evolutionary events identified: (**a**) single nucleotide level change, (**b**) small indels (≤100 bp), (**c**) large indels (>100 bp), (**d**) recombination events.

In the first instance, genetic similarities were identified between Plasmid-9, pKPC-FCF/3SP, pKPC-FCF13/05, pCF8698, pKP1433 (representing a hybrid IncN), and *bla*_KPC_ plasmids from isolates ecol_516, ecol_517, ecol_656, and ecol_736 (this study). Plasmid-9 contains duplicate Tn*4401*b elements in reverse orientation with four different 5 bp flanking sequences in an atypical arrangement within a group II intron^35^. The backbone structures of the other plasmids in this group are consistent with a separate acquisition event of a Tn*4401*b element between the *pld* and *traG* regions within an ancestral version of the Plasmid-9 structure, with the generation of a flanking TTCAG target site duplication (TSD) (labelled as Plasmid 9-like plasmid (hypothetical), Fig. 2). International spread followed by local evolution both within and across species would account for the differences between plasmids, including: (i) nucleotide level variation (observed in all plasmids); (ii) small insertion/deletion events (observed in all plasmids); (iii) larger insertion/deletion events mediated by transposable elements (e.g. pCF8698_KPC_2); and (iv) likely homologous recombination, resulting in clustered variation within a similar plasmid backbone (e.g. ecol_656/ecol_736), as well as more distinct rearrangements, including the formation of “hybrid” plasmids (e.g. pKP1433)(Fig. 2).

**Figure 3:**
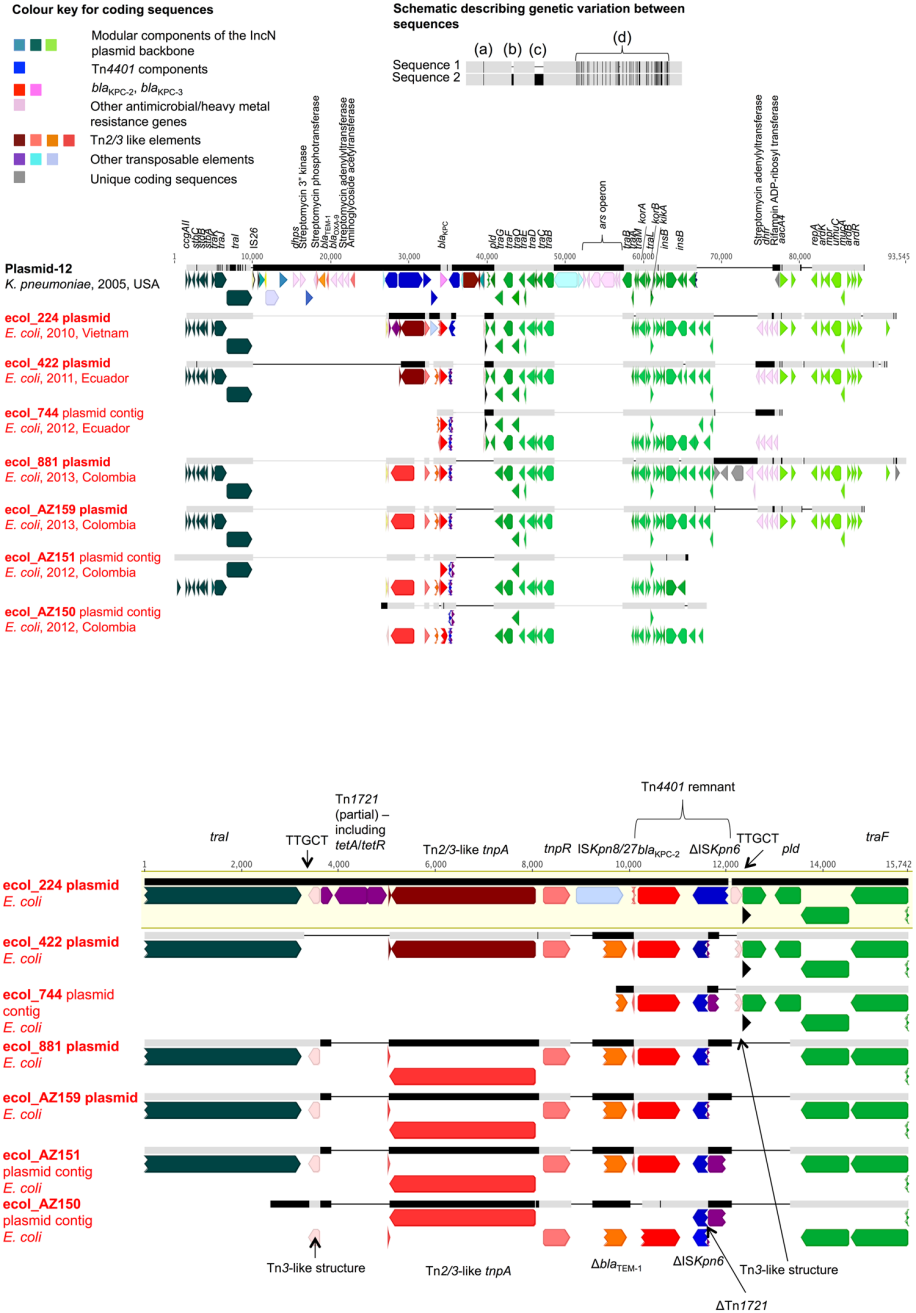
Comparison schematic of FJ223605-like (Plasmid-12-like) IncN KPC plasmids from this study. Panel 3A. Geographic origin, dates of isolation and overall alignment of plasmid/contig structures. Plasmid sequence names in red are those from this study, derived from PacBio data and closed (ecol_224, ecol_422, ecol_881, ecol_AZ159) or incomplete plasmid structures (ecol_744, ecol_AZ151, ecol_AZ150) derived from Illumina data. Aligned bars adjacent to plasmid names represent plasmid sequences: light grey denotes regions with 100% sequence homology; black represents nucleotide diversity between sequences; and thin lines represent indels. Coding sequences are represented by fat arrows below individual sequence bars and are colour-coded as per the colour key. The inset schematic describing genetic variation between sequences depicts examples of evolutionary events identified: (**a**) single nucleotide level change, (**b**) small indels (≤100 bp), (**c**) large indels (>100 bp), (**d**) recombination events. Panel 3B. Close-up of the region between *traI* and *pld* containing *bla*_KPC-2_ in study isolates only. Coding sequences are colour-coded as in Fig. 3A; sequence regions referred to in the text are annotated.

In Plasmid-12 (FJ223605), Tn*4401*b has inserted into a hybrid Tn*2*-Tn*3*-like element (with associated drug resistance genes including *bla*_TEM-1_, *bla*_OXA-9_, and several aminoglycoside resistance genes), albeit in the absence of target sequence duplication, possibly as the result of an intra-molecular, replicative transposition event generating mismatched target site sequences (L TSS = TATTA; R TSS = GTTCT). This complex is in turn located between two IS*15DIV* (IS15Δ)/IS*26*-like elements flanked by 8 bp inverted repeats, and located between the *traI* (891 bp from 3′ end) and *pld* loci (~28 Kb; Fig. 3A). The backbone components of the IncN Plasmid-12 are consistent with those seen in an NIH outbreak^5^ and in a rearranged version in a University of Virginia outbreak (CAV1043; 2008)^6^. From this study, plasmids from ecol_224, ecol_881, ecol_AZ159, ecol_422, and scaffolds from ecol_AZ151, ecol_744, ecol_AZ150 all share near identical structures to Plasmid-12, with clustered nucleotide level variation present in the *traJ*-*traI* genes, consistent with a homologous recombination event affecting this region, and evidence of sporadic insertion/deletion events (Fig. 3A). However, the *bla*_KPC-_Tn*4401* structures in these isolates are almost entirely degraded by the presence of other mobile genetic elements (MGEs), including Tn*2*/Tn*3*-like elements, IS*Kpn8/27* and Tn*1721*. In ecol_224, *bla*_KPC-2_ has been inserted into the IncN backbone as part of two repeat, inverted Tn*3*-like structures, flanked by a TTGCT TSD, and closer to *traI* (136 bp from 3′ end) than the aforementioned IS*15DIV* (IS15Δ)/IS26-like complex in Plasmid-12 (Fig. 3B). Although it is not possible to accurately trace the evolutionary history of this genomic region given the available data, the presence of shared signatures of this structure in ecol_422, ecol_744, ecol_881, ecol_AZ159, ecol_AZ150 and ecol_AZ151 suggest a common acquisition, and multiple subsequent rearrangements mediated by the presence of the large number of MGEs flanking *bla*_KPC-2_.

### Col-like plasmids may represent an important vector of transmission for *bla*_KPC_ in *E. coli*

Small col-like plasmids were the second most common type of plasmid carrying *bla*_KPC_ in *E. coli* (n = 5 [plasmids with single replicons only]), but three of these were identical (*bla*_KPC-2_, 16,559 bp), all isolated in Pittsburgh, USA, from ST131 isolates across a two year timeframe (ecol_276 [PacBio; 2010], ecol_356 [2011], ecol_875 [2013]). These three isolates additionally contained FIA, FIB, FII, X3 and X4 replicons, suggesting stable persistence of a clonal strain + plasmids over time, consistent with both SNV/core and accessory genome analyses (Fig. 1, Figure S1).

The other two col-like plasmids effectively represent short stretches of DNA encoding different mobilization genes (*mbeA*/*mbeC*/*mbeD*) harnessed to Tn*4401*/*bla*_KPC_ modules. The 5 bp sequences flanking Tn*4401* were consistent with direct, intermolecular transposition in both cases (ecol_870: TGTTT-TGTTT; ecol_867: TGTGA-TGTGA). A col/*repA* co-integrate plasmid was also observed in this dataset (ecol_AZ161), in which Tn*4401*b was inserted between colE3 signature sequences and a Tn*3* element (Tn*4401* TSS: AGATA-GTTCT). The formation of such co-integrate plasmid structures in *E. coli* has also been previously described^36^, including that of a fused col/pKpQIL-like plasmid structure (pKpQIL being historically associated with *bla*_KPC_)^37^.

Col-like plasmids have been associated with KPC-producers in other smaller, regional studies^21, 38^. Of concern, these small vectors have been shown to be responsible for the inter-species diffusion of *qnr* genes mediating fluoroquinolone resistance, even in the absence of any obvious antimicrobial selection pressure^39^. The significant association of col-like plasmids with particular *E. coli* STs (predominantly ST131) in this study could be one explanation for the disproportionate representation of *bla*_KPC_ in this lineage.

### Diverse Tn*4401* 5 bp target site sequences (TSSs) support high transposon mobility

Complete Tn*4401* isoforms flanking *bla*_KPC-2_ or *bla*_KPC-3_ were observed in only 24/43 (56%) isolates, including Tn*4401*a/a-like (n = 10; one isolate with a contig break upstream of *bla*_KPC_), Tn*4401*b (n = 12), and Tn*4401*d (n = 2) variants. Eleven different 5 bp target site sequence (TSS) pairs were identified, of which 7 (64%) were not observed in any comparison plasmid downloaded from GenBank (Table S5). Tn*4401*a had three different 5 bp TSSs, Tn*4401*b seven, and Tn*4401*d one. Most represented TSDs, but in three cases different 5 bp TSSs were flanking Tn*4401*, consistent with both direct inter- and replicative intra-molecular transposition events.

From the full set of GenBank plasmids and *in vitro* transposition experiments carried out by others, 30 different types of 5 bp TSS pairs have been characterized, seven in the experimental setting only^40^. The downloaded plasmids come from a range of species and time-points (2005–2014), although they may under-represent wider Tn*4401* insertion site diversity as a result of sampling biases. Our data however would be consistent with significant Tn*4401* mobility within *E. coli* following acquisition of diverse Tn*4401* isoforms and/or represent multiple importation events into *E. coli* from other species.

### The traditional association of *bla*_KPC_ with Tn*4401* has been significantly eroded in KPC plasmids in *E. coli*

Notably, in the other 19/43 (44%) isolates the Tn*4401* structure had been degraded through replacement with MGEs, only some of which have been previously described^41, 42^. Two isolates had novel Tn*4401*Δb structures (upstream truncations by IS*26* [ecol_270] or IS*26*-ΔIS*5075* [ecol_584]). A Tn*4401*e-like structure (255 bp deletion upstream of *bla*_KPC_) was present in three isolates (ecol_227, ecol_316, ecol_583): this was further characterized in one complete PacBio plasmid assembly (ecol_316) and represented a rearrangement at the site of the L TSS of the IS*Kpn7* element. In this plasmid, a second, partial Tn*4401* element was present without *bla*_KPC_, which would be consistent with an incomplete, replicative, intra-molecular transposition event (GGGAA = L TSS and R TSS on the two Tn*4401*b elements, in reverse orientation). Other motifs flanking *bla*_KPC_ included: hybrid Tn*2*/Tn*3* elements-IS*Kpn8/27*-*bla*_KPC_ (n = 1; ecol_224); IS*26*-Δ*tnpR*(Tn*3*)-IS*Kpn8/27*- *bla*_KPC_-ΔTn*1721*-IS*26* (n = 5; ecol_AZ153-AZ155, ecol_AZ166, ecol_AZ167); IS*Apu2*-*tnpR*(Tn*3*)-Δbla_TEM_ -*bla*_KPC_- *korC*-*klcA*-ΔTn*1721*-IS*26* (n = 1; ecol_542); IS*26*-*tnpR*(Tn*3*)-Δbla_TEM_ -*bla*_KPC_-korC-IS*26* (n = 1; ecol_545); hybrid Tn*2*/Tn*3* elements + Δbla_TEM_-*bla*_KPC_-ΔTn1721 (n = 2; ecol_744, ecol_422), Tn*3* elements-Δ*bla*_TEM_-*bla*_KPC_- ΔTn*1721* (n = 4; ecol_881, ecol_AZ151, ecol_AZ159, ecol_AZ150) and ΔTn*3*-Δ -ΔIS*3000* (Tn*3*-like) (n = 1; ecol_AZ152). We were unable to assess the flanking context of *bla*_KPC_ in ecol_452 due to limitations of the assembly.

This apparent diversity in independently acquired MGEs around the *bla*_KPC_ gene extends the means by which *bla*_KPC_ can be mobilized. Interestingly, as observed previously^43^, all the degraded Tn*4401* sequences in this dataset were associated with variable stretches of flanking Tn*2*/*3*-like sequences, suggesting that the insertion of Tn*4401* into a Tn*2*/Tn*3*-like context may have enabled the latter to act as a hotspot for the insertion of other MGEs^6^. A particular finding of note is the association with IS*26*, which has been linked to the dissemination of several other resistance genes in *E. coli*, including CTX-M ESBLs^24, 44^; is able to increase the expression of closely co-located resistance genes^45^; participates in co-integrate formation and hence plasmid rearrangement^46^; and enhances the occurrence of other IS*26*-mediated transfer events into plasmids harbouring IS*26* ^46^.

## Discussion

This study of KPC-*E. coli* obtained from two global resistance surveillance schemes has demonstrated the diversity of genetic structures associated with *bla*_KPC_ at all genetic levels, including: (i) host bacterial strain; (ii) plasmid types; (iii) associated transposable MGEs, including transposons and insertion sequences; and (iv) *bla*_KPC_ alleles. This has previously been observed within institutional, poly-species outbreaks, particularly for non-*E. coli* Enterobacteriaceae^5, 6^, as well as in a more recent study of nine KPC-*E. coli* from the US^47^. We have identified global and regional *bla*_KPC_ spread at the strain and plasmid levels, including signatures consistent with inter-species spread of plasmids, over short timeframes. Although the geographic reach of sampling has been more substantial than any other similar study, there are some limitations in the sampling consistency of both surveillance schemes^22^ (e.g. isolates from China were only submitted in 2008, 2012 and 2013).

We utilized long-read sequencing on only a subset of isolates, given resource limitations, allowing us to completely resolve chromosomal and plasmid structures in less than half the isolates. Nevertheless, despite this drawback, we have highlighted the extraordinary diversity present. This study, along with other recent analyses utilizing long-read sequencing to resolve antimicrobial resistance plasmids^5, 6^, also demonstrates the difficulty in making evolutionary comparisons for MGEs, given the absence of effective phylogenetic methods/tools to characterize their genetic histories which commonly involve genetic rearrangements, and evolutionary events that are not restricted to single nucleotide mutations.

This study has demonstrated the particular association of *bla*_KPC_ in *E. coli* with IncN plasmids, previously associated with the spread of other antimicrobial resistance elements^48^, as well as col-like plasmids, which are small, potentially highly mobile, and generally high copy-number units. The traditional association of *bla*_KPC_ with Tn*4401* has apparently been eroded in *E. coli*, with the complete Tn*4401* structure absent in 50% of strains investigated. This finding is in contrast to most global descriptions of *K. pneumoniae* where *bla*_KPC_ has been stably associated with largely intact Tn*4401* isoforms for more than a decade. Instead, other shorter MGEs, such as Tn*2*/Tn*3*-like elements and IS*26*, appear to be commonly involved in *bla*_KPC_ dispersal in *E. coli*. These MGEs have been associated with the spread of multiple resistance mechanisms, such as *bla*_TEM_ and *bla*_CTX-M_, and will potentially similarly contribute to the dissemination of *bla*_KPC_ in *E. coli*. We did not undertake any functional assays investigating the dynamics of *bla*_KPC_ transmission in *E. coli* to support this hypothesis, but this would be illuminating work for future study.

The global emergence and spread of *bla*_KPC_ in *E. coli* has been driven by multiple mechanisms, including local and international spread of highly genetically related strains, exchange of plasmids with other Enterobacteriaceae and between *E. coli* lineages, transposition events within the species, and a breakdown of the traditional association of *bla*_KPC_ with Tn*4401*. The genetic flexibility observed is impressive, especially given the timeframes and number of KPC-*E. coli* characterized. Tracking the spread of resistance genes given such multi-level genetic variability is complicated, even with a high-resolution typing method such as WGS. The association of *E. coli*, both a common pathogen and commensal in a wide range of environmental/animal reservoirs, with MGEs (col-like plasmids, IS*26*) that have been shown to facilitate the dissemination of other successful resistance genes even in the absence of antimicrobial selection pressures, may represent a difficult situation to control.

## Methods

### Isolate collection and sampling frames

Isolates were obtained from two global antimicrobial resistance surveillance schemes (The Merck Study for Monitoring Antimicrobial Resistance Trends [SMART], 2008–2012; AstraZeneca global surveillance study of antimicrobial resistance, 2012–2013; 417 institutions operating in 95 countries), as previously described^22^. Of 55,874 isolates collected, 45 (0.08%) were positive for *bla*_KPC_ by PCR (n = 7 from 2010, 10 from 2011, 13 from 2012, 15 from 2013; Table S1). Isolates had been previously characterized using partial, sequenced-based typing methods, including multi-locus sequence typing (MLST; Achtman scheme), *fimH* typing, PCR for beta-lactamases, strain/plasmid PFGE (Table S1)^22^.

### DNA extraction and sequencing

All isolates were sequenced on the Illumina MiSeq; a subset of 18 were purposively selected for PacBio sequencing, to capture potential diversity across a range of years of isolation, geographic location, standard ST, plasmid size and resistance gene content (based on laboratory typing). DNA for sequencing was extracted from sub-cultures of bacterial stocks (frozen at −80 °C; cultured overnight on Columbia blood agar at 37 °C) using the Qiagen Genomic tip 100/G extraction kit, as per the manufacturer’s instructions (Qiagen, Hilden, Germany; catalogue no: 10243).

DNA libraries for MiSeq sequencing were generated and normalized using 300 base, paired-end Nextera XT DNA library preparation kits (Illumina, San Diego, CA, USA). PacBio sequencing on the subset of strains was performed as previously described^49^; in these cases, the same DNA extract was used for both Illumina and PacBio sequencing approaches.

### Sequence data processing

#### Illumina (short-read data)

Mapping-based approaches: Prior to reference-based mapping to the O150:H5 SE15 *E. coli* reference genome (Genbank accession: NC_013654), Illumina data were trimmed using cutadapt version 1.5. SE15, which is ST131, was chosen as the reference given the largest number of strains sequenced (and in the dataset) came from this ST. Repetitive regions of the reference were identified using self-self BLASTn analysis with default settings; these regions were then masked prior to mapping and base calling. Properly paired sequence reads were mapped to the reference using Stampy (v1.0.17) (Supplementary methods).

Single-nucleotide variants (SNVs) were determined across all mapped non-repetitive sites using SAMtools (version 0.1.18) mpileup. mpileup was run twice to separate high-quality base calls from low-quality base calls; variant call format (VCF) files of annotated variant sites were created using GATK (v1.4.21). Base calls derived from these two VCF files were filtered to retain only high quality calls (Supplementary methods).

Core variable sites (site called in all sequenced isolates, excluding “N” or “-” calls) derived from mapping to the SE15 reference were “padded” with invariant sites in a proportion consistent with the GC content and length of the reference genome (4.72 Mb, 51% average GC content), to generate a modified alignment of input sequences to generate phylogenies. Phylogenies were reconstructed using RaxML (Version 7.7.6)^50^, with a generalized time reversible model, four gamma categories (allowing for variable rates of mutation between sites), and bootstrapped 100 times.

De novo assemblies of Illumina data: *De novo* assemblies of short-read Illumina data for all isolates were generated using the A5-MiSeq pipeline (version 20140604; default settings)^51^, which includes adapter/low-quality region read trimming steps (Trimmomatic), initial contig assembly, crude scaffolding, misassembly correction and final scaffolding. We used the unscaffolded contigs file in subsequent analyses (*.contigs.fasta).

### PacBio (long-read data)

DNA library preparation for and sequencing on the PacBio RSII were performed in accordance with the manufacturer’s instructions, using P5-C3 sequencing enzyme and chemistries respectively, and following a 7–50 kbp fragment selection step (full details in ref. 49). *De novo* assemblies were constructed using HGAP3 (version 2.2.0)^52^, resulting in phased chromosomal and plasmid contigs, which were manually closed by resolving and trimming overlapping repeats at the contig ends. Illumina reads for the respective isolate were then mapped to the resulting closed PacBio assemblies using bwa-MEM (version 0.7.9a-r786, default settings)^53^. Read pileups were visualized in Geneious^54^; mismatches between the sequence derived from mapping and the reference PacBio assemblies were inspected manually to identify the correct structure. Finally, to capture small plasmids that may have been filtered out due to size-selection of DNA fragments >7 kbp prior to PacBio sequencing, unmapped Illumina reads (extracted using the SAMtools view command, with the -f 12 flag) derived from this process were *de novo* assembled using the A5-MiSeq pipeline 20140604^51^; any assembled contigs were manually closed by assessing for and trimming overlapping repeats (100% match over a length of ≥100 bp; no match to any other contig in the assembly). The final consensus chromosomal and plasmid sequences derived from these processes were used in analyses and submitted to GenBank.

### Automated and manual annotation of *de novo* assemblies

All plasmid structures and *de novo* assemblies were annotated using PROKKA^55^, with subsequent manual refinement of annotations for regions of interest using BLASTn^56^ and the NCBI bacterial and ISFinder databases^57^. Alignments of sequence structures were visualized and modified in Geneious. *bla*_KPC_-containing contigs identified in the *de novo* assemblies derived from Illumina data were manually inspected for overlapping repeats as above; if these were present, these contigs were considered additional putative KPC plasmids and trimmed and closed as above.

### Core/accessory genome comparisons

These were undertaken using the pangenome pipeline, ROARY^27^, by inputting the *.gff files generated from the PROKKA annotation of each of the Illumina *de novo* assemblies (default settings). Comparisons were made separately for all isolates and the ST131 subset. The output gene_presence_absence.csv files were processed using the pheatmap function in R. Resistance genes were identified using ResistType, a command-line tool developed in-house that identifies the presence of reference loci in WGS data using both BLASTn against *de novo* assemblies and mapping-based approaches, and estimates copy number by comparing contig coverage at any given locus with median contig coverage [scripts, reference resistance gene database and manual available at: https://github.com/hangphan/resistType]. These features were plotted on the maximum likelihood phylogenies using the Ape package in R.

### Comparisons with publicly available KPC plasmid sequences

All complete KPC RefSeq plasmid sequences available in GenBank in May 2015 were identified using the search terms “plasmid” + “KPC” + “complete sequence”. The resulting list was filtered manually to exclude any additional sequences present that were not complete plasmid sequences. In total 63 plasmid sequences were included (Table S5).

For the IncN plasmid comparisons, we included the following from our dataset: (i) six cases where PacBio sequencing had fully resolved the *bla*_KPC_ IncN plasmid (ecol_224, ecol_422, ecol_517, ecol_656, ecol_881, ecol_AZ159); (ii) two cases where the IncN rep and *bla*_KPC_ were co-located on the same, incomplete contig (ecol_516, ecol_736); and (iii) three cases where *bla*_KPC_ was present in isolates containing an IncN rep and on contigs that showed high similarity to the IncN plasmid backbones under scrutiny (ecol_744, ecol_AZ150, ecol_AZ151).

### Availability of Data and Materials

Sequencing datasets (Illumina raw reads, PacBio assemblies) are available in GenBank/SRA (project accession: PRJNA316786 (https://www.ncbi.nlm.nih.gov/bioproject/?term=316786)(Table S1).

## Electronic supplementary material

Supplementary material

Table S1

Table S2

Table S3

Table S4

Table S5
