# Supplementary material for: Genomic epidemiology of global Klebsiella pneumoniae carbapenemase (KPC)-producing Escherichia coli
Source: Sci Rep. 2017 Jul 19;7:5917. doi: 10.1038/s41598-017-06256-2 (PMC5517641; doi:10.1038/s41598-017-06256-2)
Supplement: Supplementary file 1 — Supplementary material [file 41598_2017_6256_MOESM1_ESM.pdf]

SUPPLEMENTARY MATERIAL

Manuscript title:

Genomic epidemiology of global *Klebsiella pneumoniae* carbapenemase (KPC)-producing *Escherichia coli*

Author list:

Stoesser N, Sheppard AE, Peirano G, Anson LW, Pankhurst L, Sebra R, Phan HTT, Kasarskis A, Mathers AJ, Peto TEA, Bradford P, Motyl MR, Walker AS, Crook DW, Pitout JD

Supplementary methods

Illumina read processing

Stampy

Run without Burrows-Wheeler Aligner premapping, using an expected substitution rate of 0.01

cutadapt

This was run specifying the adapter sequence using "-a CTGTCTCTTATACACATCT"

mpileup flags

- first run, with options "-E -M0 -Q25 -q30 -m2 -D -S" and otherwise default settings
- second run, with options "-B -M0 -Q0 -q0 -m2 -D -S" and otherwise default settings

Base call filtering

Only those calls passing the following filters were retained:

(i) the proportion of high-quality bases supporting the call was ≥90%, and ≥5 high-quality bases were required as a minimum, with at least one in the forward direction and at least one in the reverse direction

(ii) the root of the mean square mapping quality of reads covering the site was ≥30

(iii) the Phred scaled quality supporting the call was ≥25

(iv) reads spanning the site were made up of ≥35% high-quality bases

(v) the site was not called as heterozygous

Comparisons of A5-MiSeq and SPAdes assemblies for isolates sequenced by both PacBio and Illumina in this dataset (n=18).

The closed PacBio assembly for each isolate was used as the respective reference for comparisons.

1. Total number of contigs

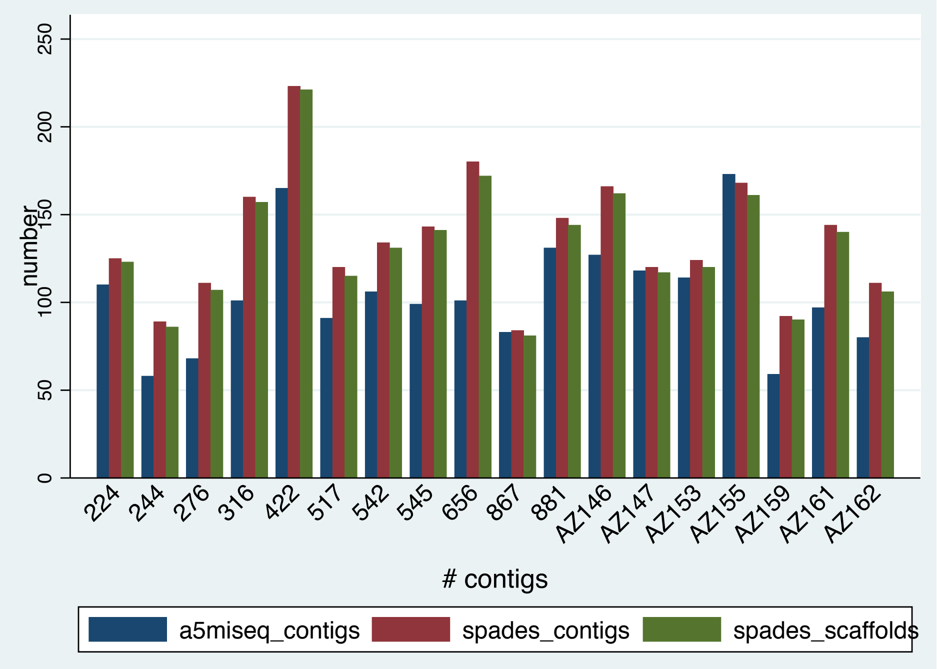

2. N50

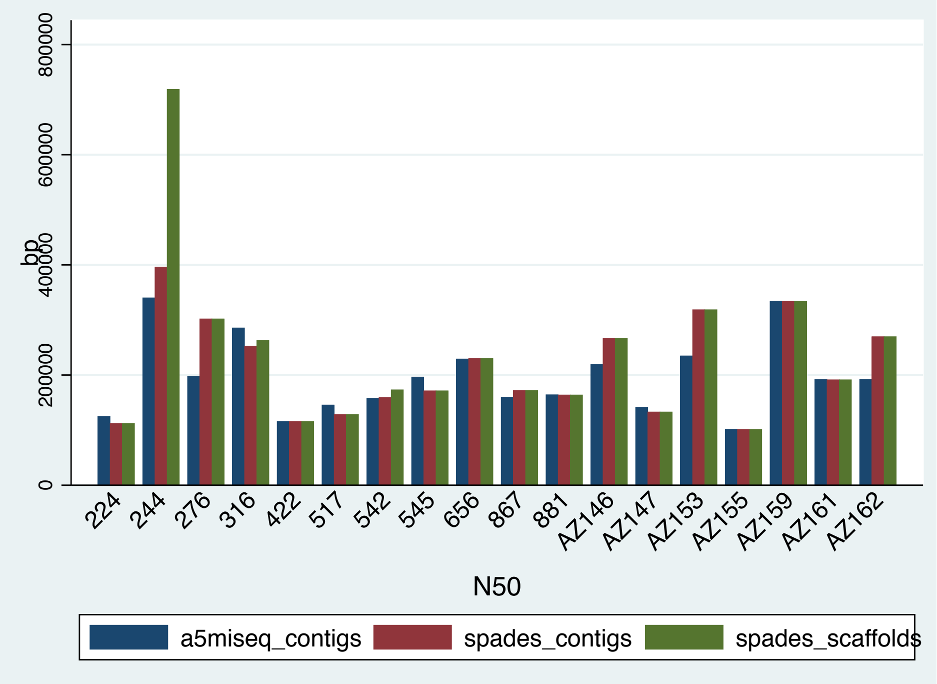

3. Percentage of reference captured following alignment of contigs to reference

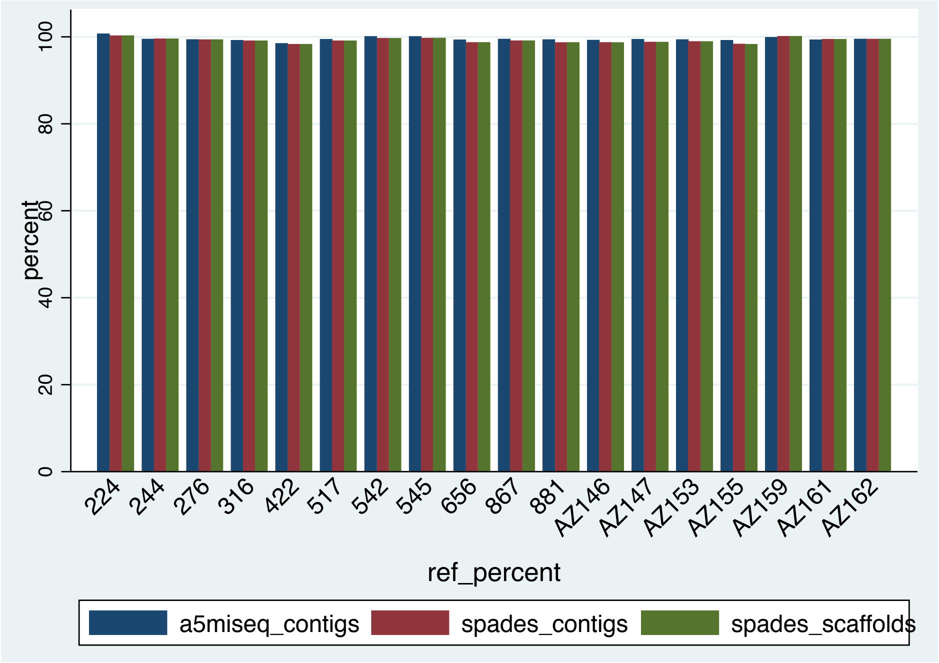

4. Comparisons of *de novo* assembled KPC *E. coli* isolates using unscaffolded A5-MiSeq (top) and SPAdes contigs (bottom), versus the PacBio assembly for each respective isolate (chromosome plus plasmids). Each assembled contig is represented by a coloured block, staggered to represent contig boundaries. Contigs are coloured (i) green if they are consistent with the reference and (ii) red if they contain misassemblies.

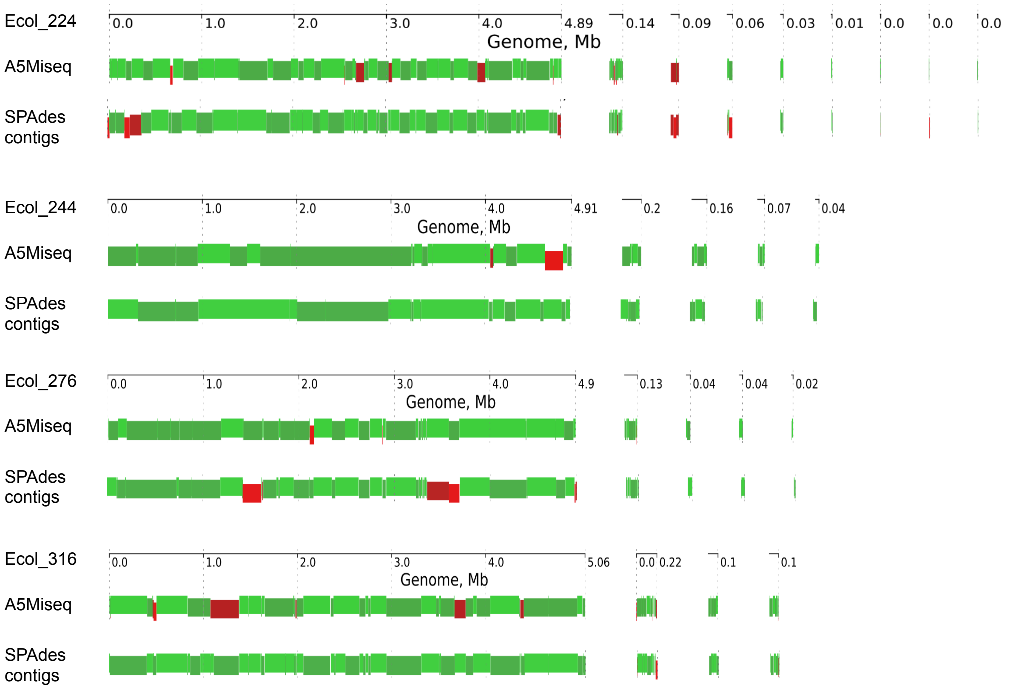

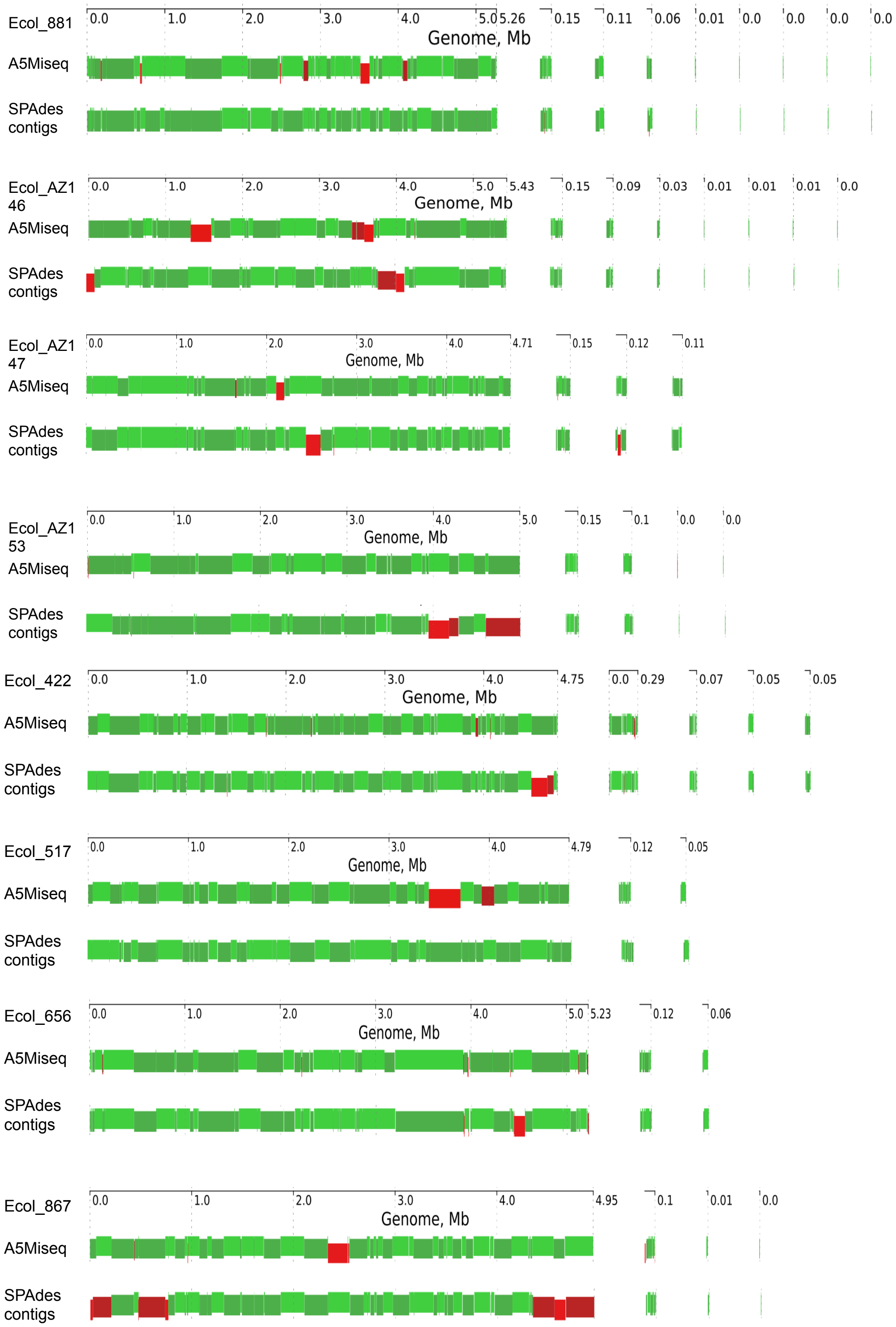
Ecol_881
A5Miseq
SPAdes contigs
Genome, Mb
Ecol_AZ146
A5Miseq
SPAdes contigs
Genome, Mb
Ecol_AZ147
A5Miseq
SPAdes contigs
Genome, Mb
Ecol_AZ153
A5Miseq
SPAdes contigs
Genome, Mb
Ecol_422
A5Miseq
SPAdes contigs
Genome, Mb
Ecol_517
A5Miseq
SPAdes contigs
Genome, Mb
Ecol_656
A5Miseq
SPAdes contigs
Genome, Mb
Ecol_867
A5Miseq
SPAdes contigs
Genome, Mb

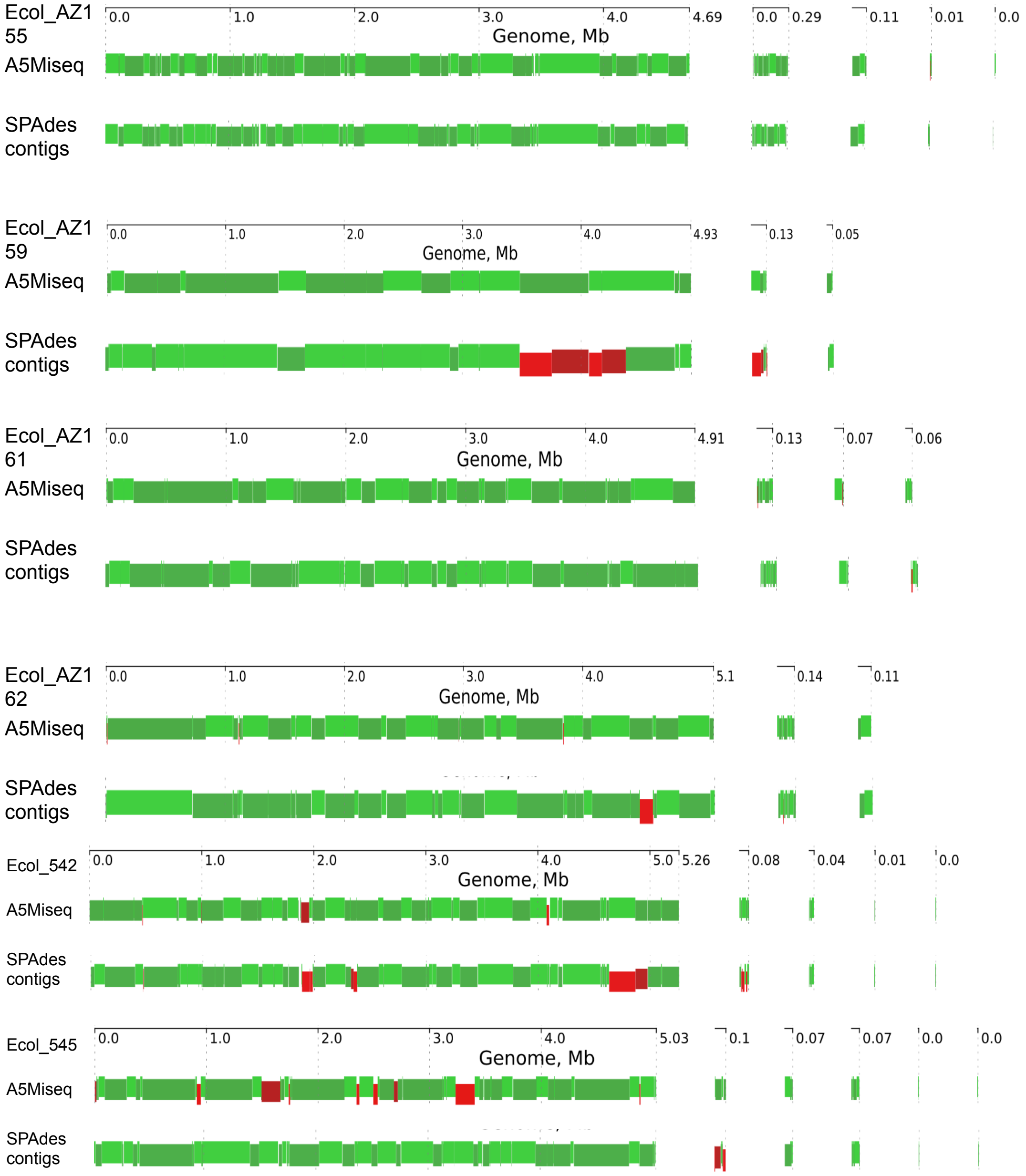

Ecol_AZ155
A5Miseq
SPAdes contigs
0.0
1.0
2.0
3.0
4.0
4.69
0.0
0.29
0.11
0.01
0.0
Genome, Mb
Ecol_AZ159
A5Miseq
SPAdes contigs
0.0
1.0
2.0
3.0
4.0
4.93
0.13
0.05
Genome, Mb
Ecol_AZ161
A5Miseq
SPAdes contigs
0.0
1.0
2.0
3.0
4.0
4.91
0.13
0.07
0.06
Genome, Mb
Ecol_AZ162
A5Miseq
SPAdes contigs
0.0
1.0
2.0
3.0
4.0
5.1
0.14
0.11
Genome, Mb
Ecol_542
A5Miseq
SPAdes contigs
0.0
1.0
2.0
3.0
4.0
5.0
5.26
0.08
0.04
0.01
0.0
Genome, Mb
Ecol_545
A5Miseq
SPAdes contigs
0.0
1.0
2.0
3.0
4.0
5.03
0.1
0.07
0.07
0.0
0.0
Genome, Mb

5A. Assessment of KPC-harbouring contigs for each assembler - size and dotplot (sequence alignment characteristics)

**Isolate**	**A5-Miseq kpc contig size (bp)**	**SPAdes kpc contig size (bp)**	**Size difference** (positive value indicates A5-Miseq contig is bigger, negative value indicates SPAdes contig is bigger)
ecol_224	38355	49718	-11363
ecol_244	24191	23851	340
ecol_276	16858	16686	172
ecol_316	6066	5722	344
ecol_422	19735	19391	344
ecol_517	39072	35306	3766
ecol_542	43620	25825	17795
ecol_545	7032	3045	3987
ecol_656	47373	56497	-9124
ecol_867	14328	14052	276
ecol_881	33687	32099	1588
ecol_AZ146	42505	45138	-2633
ecol_AZ147	63239	62896	343
ecol_AZ153	7483	7142	341
ecol_AZ155	7485	7142	341
ecol_AZ159	47531	47658	-127
ecol_AZ161	11170	10259	911
ecol_AZ162	12197	11900	297

5B. Dotplot comparisons of KPC-harbouring contigs by A5-Miseq and SPAdes assemblers can be accessed in the zipped datasets included as part of the online supplement.

SUPPLEMENTARY FIGURE

Supplementary Figure S1. Phylogeny for the ST131 KPC-*Escherichia coli* strains identified in this study (n=16), and heatmap representing associated core and accessory components.

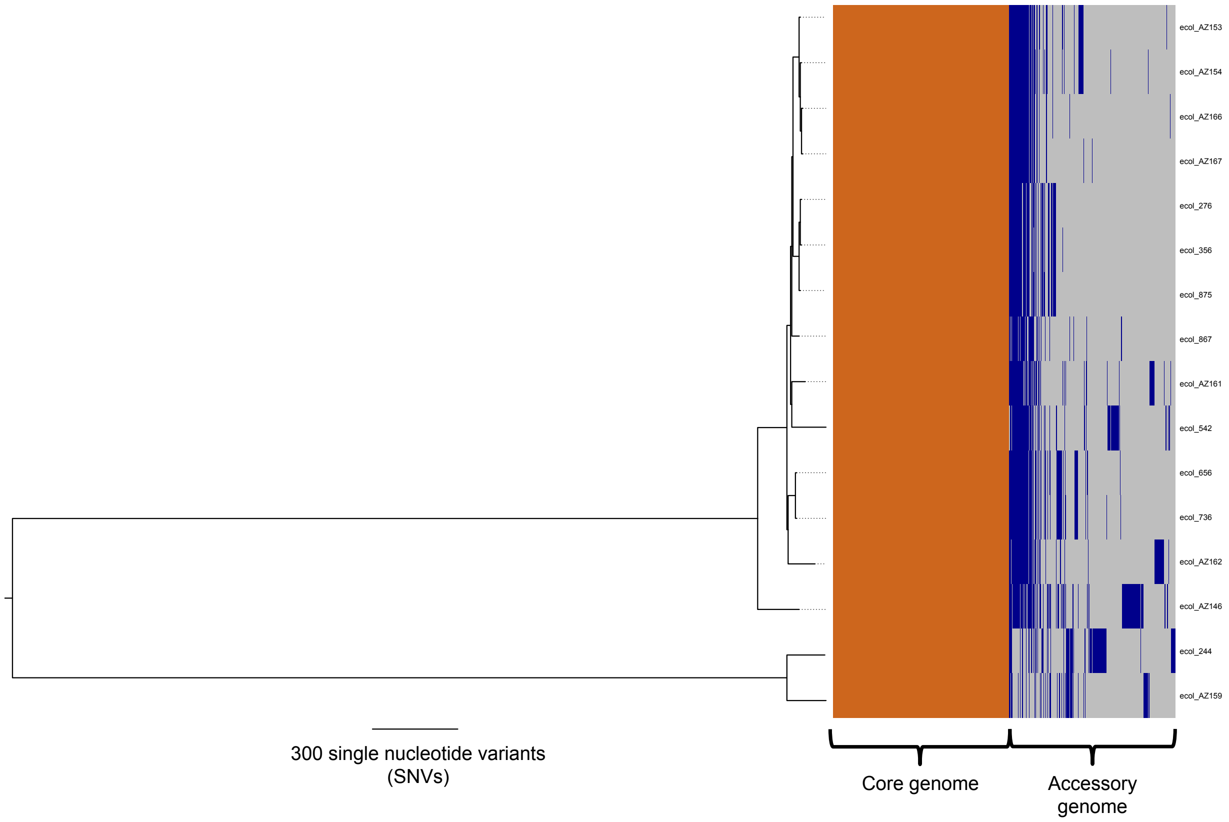

SUPPLEMENTARY TABLES (included as separate files)

Supplementary Table 1. Original laboratory-based typing results for study strains, sequencing methods used to generate whole genome sequencing (WGS) data for the study, WGS quality metrics, and WGS-based typing results.

Supplementary Table 2. Information on the presence, copy number and nucleotide/amino acid level variation of resistance genes identified from the WGS data using the ResistType tool. Also includes typing results for the sequenced DH10B *Escherichia coli* control, and for the ecol_252 isolate which was excluded from subsequent analyses as bla_{KPC} was not present in the sequenced extract.

Supplementary Table 3. Details of top blast matches of each of the fully resolved plasmid sequences identified in this study.

Supplementary Table 4. Publicly available IncN bla_{KPC} plasmid sequences used for comparisons.

Supplementary Table 5. Publicly available plasmid sequences used for comparisons.
