# Supplementary material for: Genomic epidemiology of global Klebsiella pneumoniae carbapenemase (KPC)-producing Escherichia coli
Source: Sci Rep. 2017 Jul 19;7:5917. doi: 10.1038/s41598-017-06256-2 (PMC5517641; doi:10.1038/s41598-017-06256-2)
Supplement: Supplementary file 5 — Table S4 [file 41598_2017_6256_MOESM5_ESM.doc]

**Supplementary Table 4.** Publicly available IncN *bla*_KPC_ plasmid sequences used for comparisons.

| Plasmid name | Host strain | Location | Time-point | Species | Sequence type | Size (bp) | *bla*_KPC_ variant | Tn4401 | L IRR | R IRR | Accession number |
| --- | --- | --- | --- | --- | --- | --- | --- | --- | --- | --- | --- |
| Plasmid 9 | Strain 9 | New York City, New York, USA | 2005 | *Klebsiella pneumoniae* | Unknown | 70,655 | 2 (duplicate) | b | ATTTT  TGGTA | TGGTA  TTGAA | FJ223607 |
| Plasmid 12 | Strain 12 | New York City, New York, USA | 2005 | *K. pneumoniae* | Unknown | 75,617 | 2 | b | TATTA | GTTCT | FJ223605 |
| pKPC-FCF13/05 | FCF1305 | Sao Paulo state, Brazil | May 2005 | *K. pneumoniae* | 442 | 53,081 | 2 | b-like (insertion) | TTCAG | TTCAG | CP004366 |
| pBK31551 | BK31551 (CK7) | New Jersey, USA | September 2005 | *K. pneumoniae* | 834 | 83,712 | 4 | Large insertion in Tn*4401* | TTTCA | AAAGC | JX193301 |
| pKPC-CAV1043 | CAV1043 | Charlottesville, Virginia, USA | March 2008 | *Enterobacter asburiae* | Unknown | 59,138 | 3 | b | GTTCT | GTTCT | CP011589.1 |
| pYD626E | YD626E | Pittsburgh, Pennsylvania, USA | 2008-2011 | *Escherichia coli* | 648 | 72,800 | 2 | b | GTTCT | GTTCT | KJ933392 |
| pKPC-FCF/3SP | FCF3SP | Sao Paulo state, Brazil | September 2009 | *K. pneumoniae* | 442 | 54,605 | 2 | b | TTCAG | TTCAG | CP004367 |
| pKP1433 | KP1433 | Athens, Greece | 2009-2010 | *K. pneumoniae* | 340 | 55,417 | 2 | b-like (truncated at position 611) | - | TTCAG | JX397875 |
| pKPC-47e | ECNIH3 | Bethesda, Maryland, USA | September 2011 | *Enterobacter cloacae* | 97 | 50,333 | 2 | - | - | GTTCT | CP008901 |
| pKPC-860 | ECNIH4 | Bethesda, Maryland, USA | November 2012 | *E. cloacae* | 191 | 56,557 | 2 | - | - | GTTCT | CP009853 |
| pKo6 | Unknown | Fudan, China | Earlier than April 2013 | *Klebsiella ozaenae* | Unknown | 66,549 | 2 | - | - | - | KC958437 |
| pKPC-1c5 | PSNIH1 | Bethesda, Maryland, USA | March 2013 | *Pantoea* spp. | Unknown | 65,549 | 2 | - | - | GTTCT | CP009881 |
| pKPC_SMH | GN1006 | Toronto, Ontaria Canada | March-May 2013 | *K. pneumoniae* | Unknown | 53,262 | 3 | b | ATGCA | ATGCA | KT148595 |
| pKPC-629 | ECONIH1 | Bethesda, Maryland, USA | September 2013 | *E. coli* | 648 | 80,186 | 2 | b | GTTCT | GTTCT | CP009862 |
| pKPC-e4e | KPNIH29 | Bethesda, Maryland, USA | November 2013 | *K. pneumoniae* | 1518 | 62,589 | 3 | b | GTTCT | GTTCT | CP009864 |
| pCF8698_KPC2 | CF8698 | South Hessen, Germany | January-June 2014 | *Citrobacter freundii* | Unknown | 54,036 | 2 | Truncated at position 7,173 | - | TTCAG | LN610760 |
